# Diagnostic utility of serum and pleural levels of adenosine deaminase 1–2, and interferon-γ in the diagnosis of pleural tuberculosis

**DOI:** 10.1186/2049-6958-9-12

**Published:** 2014-03-06

**Authors:** Sibel Yurt, Canan Küçükergin, Burcu Arpinar Yigitbas, Şule Seçkin, Hüseyin Cem Tigin, Ayşe Filiz Koşar

**Affiliations:** 1 Department of Chest Diseases, Yedikule Chest Disease and Surgery Training and Research Hospital, Istanbul, Turkey; 2 Department of Clinical Biochemistry, Istanbul Medical Faculty, Istanbul University, Istanbul, Turkey

**Keywords:** Adenosine deaminase 1-2, Exudative pleural effusion, Interferon gamma, Pleural tuberculosis

## Abstract

**Background:**

To evaluate and compare the diagnostic efficiency of serum (s) and pleural (p) levels of adenosine deaminase (ADA)-1, ADA-2, total ADA, and interferon-gamma (IFN-γ) for the differential diagnosis of pleural tuberculosis (TB).

**Methods:**

Clinical and analytic data of 93 consecutive patients with pleural effusions from May 2012 to February 2013 were prospectively evaluated. The study population included 43 pleural TB, 23 malignancies, and 27 other exudates. The median and interquartile range of ADA-1, ADA-2, total ADA, and IFN-γ were evaluated according to their underlying diseases.

**Results:**

There were no significant differences in sADA-1 and sIFN-γ values among each group. pADA-1, pADA-2, total pADA, and pIFN-γ levels were significantly higher in patients with pleural TB than in other patients (p < 0.0001). As for pleural TB receiving operating characteristic (ROC) curves identified the following results. The best cut-off value for pADA-2 was 20.37 U/L and it yielded a sensitivity and specificity of 95.35% and 86%, respectively. Taking a cut-off value of 40.68 U/L for total pADA, the sensitivity and the specificity were found to be 88.37% and 88%, respectively. ROC curve identified 110 U/L as the best cut-off value for pμg/ml, while the sensitivity and the specificity were 74.42% and 68%, respectively. Finally, the best cut-off value for pADA-1 was 16.8 U/L and yielded a sensitivity and specificity of 69.77% and 68%, respectively.

**Conclusions:**

To distinguish pleural TB, pleural levels of ADA-2 have the highest sensitivity among the different diagnostic parameters and may find a place as routine investigation for early detection of TB in the future.

## Background

Pleural effusion (PE) is a very common clinical problem and a manifestation of extrapulmonary tuberculosis (TB). It may occur in patients affected with pulmonary TB, pneumonia, malignancy, congestive cardiac failure, cirrhosis of liver, nephrotic syndrome, pulmonary infarction and connective tissue disorders [1]. TB is one of the most common causes of PE especially in the areas of high prevalence. Diagnosis of TB pleurisy is based on observation of bacilli in pleural fluid or granulomas in pleural biopsy specimens. Because of the rarity of bacilli in pleural fluid, only 5% of the effusions are smear positive for acid-fast bacilli and only 25% to 37% of the effusions grow Mycobacterium tuberculosis on the cultures [2].

Several biological markers have been proposed to enhance the effectiveness of the diagnosis of pleural TB. Adenosine deaminase (ADA) is an enzyme involved in the conversion of adenosine to inosine. Two isoenzymes of ADA have been identified. They are known as ADA-1 and ADA-2. ADA-1 is present in nearly all body cells. ADA-2 is mainly expressed in monocytes and macrophages and is released to the extracellular space following stimulation of these cells triggered by intracellular infection [3]. A number of studies have analysedADA and its isoenzymes in pleural effusions, and have found that ADA-2 isoenzyme is primarily responsible for the total ADA activity in tuberculous effusions, while ADA-1 is the major isoenzyme in parapneumonic effusions [2,3]. Equally, the highest ADA activity was found in lymphocytes and monocytes. ADA-2 could be detected only in monocytes and serum ADA most probably originated from lymphocyte precursors. Valdes et al. demonstrated that the activity of both isoenzymes contributes to the high ADA activity in pleural TB, with ADA-2 playing the predominant role [4]. Elevated ADA activity is not specific for pleural TB, but is also seen in parapneumonic effusions and pleural empyemas, rheumatoid effusions, and certain malignant pleural effusions. Another highly sensitive and specific marker of the pleural TB is interferon-gamma (IFN-γ) [5]. The increased IFN-γ concentration in TB can be explained by the cellular mechanisms of immune reactions to infection with *M. tuberculosis*. Expression of *M. tuberculosis* antigens on the surface of antigen-presenting cells leads to T cell activation, increased T cell count, and release of cytokines that play a role in the further stages of the immune response [6].

The aim of the present study was to evaluate and compare the diagnostic efficiency of serum (s) and pleural (p) levels of ADA-1, ADA-2, total ADA, and IFN-γ for the differential diagnosis of pleural TB.

## Methods

### Study design

The study was approved by the Ethical Committee of the Yedikule Chest Disease and Chest Surgery Education and Research Hospital. Written informed consent was obtained from all subjects. Clinical and analytic data of 93 consecutive patients, admitted to our tertiary center with pleural effusions and diagnosed by analysis of fluid samples obtained by thoracentesis or chest drainage from May2012 to February 2013, was prospectively evaluated.

All patients provided a medical history, and underwent detailed physical examination and routine laboratory tests. Flexible bronchoscopy, computed tomography of the chest, and echocardiogram were performed when indicated. Pleural effusions were defined as exudates when the analysis indicated that the fluid satisfied the criteria of Light et al. [7]. Cytological and microbiological examinations of pleural fluid were performed. All tuberculosis patients were diagnosed with pleural biopsy. Pleural effusions were diagnosed as malignant when fluid cytology or pleural biopsy findings were positive for malignancy. All other exudative effusions were included in the miscellaneous group. The patients who had not met the above mentioned diagnostic criteria for pleural effusion and also those who had been diagnosed with the heart failure, kidney failure, liver cirrhosis, and HIV infection, those on TB treatment, those with empyemas, and those with nephrotic syndrome and transudate were not included in the study. Furthermore, patients affected with pleural effusions of unknown etiology were excluded from the study.

The median and interquartile range of ADA-1, ADA-2, total ADA, and IFN-γ levels in the serum and PE were evaluated according to their underlying diseases for the diagnostic accuracy.

### Measurement of serum and pleural adenosine deaminase levels

ADA activity in the pleural fluid was determined with De Giusti’s method and the results were recorded as IU/L in all the patients [8]. A blood sample was taken from the antecubital vein, which was then centrifuged at 3000xg for 10 min. within half an hour and stored at -20°C until assay. The manual kinetic ADA activity assay was optimized for the automated analyzer (Konelab 60 I, Thermo Labsystems CLD, Espoo, Finland). For the determination of ADA activity, the ammonia produced by the enzymatic activity was coupled to 2-oxoglutarate by glutamate dehydrogenase. 2-oxoglutarate was activated by adenosine diphosphate (ADP). In this reaction, NADH was used as indicator and the reaction was followed by the decrease of absorbance at 340 nm, according to method developed by Ellis [9]. To distinguish ADA-1 from ADA-2, the activity was measured using the same techniques with Erythro-9 (2-hydroxy-3-nonyl) adenine (EHNA) which is a potent inhibitor of only ADA-1 isoenzyme, showing the ADA-2 activity. The activity of ADA-1 was calculated by subtracting the ADA-2 activity from total ADA activity.

### Measurement of serum and pleural interferon-γ levels

The second generation QuantiFERON^®^ TB-Gold test (Cellestis^®^, Carnagie Australia) measures *in vitro* IFN-γ production originating from immune T-cells during *in vitro* stimulation with peptides of the *M. tuberculosis* specific antigens [10,11]. One ml of blood sample or 1 ml of pleural fluid mononuclear cells isolated from pleural fluid by density gradient centrifugation was added to QuantiFERON^®^-TB Gold (QFT-TB) tubes. The tubes were centrifuged and 500 μl of the supernatants were harvested and stored at -70°C until the IFN-γ was measured in an ELISA reader. The μg/ml concentrations (IU/ml) were calculated by the ‘QFT-TB analysis Software’.

### Statistical analyses

Data were analysed using the Statistical Package for Social Sciences (SPSS) software version 19.0 (SPSS Inc., Chicago, IL) and Medcalc (Mariakerke, Belgium) for Windows. A normal distribution of the quantitative data was checked using Kolmogorov-Smirnov test. Parametric tests were applied to data of normal distribution and non-parametric tests were applied to data of questionably normal distribution. Mann–Whiney *U*-test was used to compare two independent groups. The Kruskal–Wallis analysis of variance test was used to compare groups and the Bonferroni-corrected Mann–Whitney *U*-test was used as a more conservative measure of significance for multiple comparisons. Receiver operating characteristic (ROC) curves were used to identify the optimal cut-off points. The distribution of categorical variables in both groups was compared using Pearson chi-square test. Data were expressed as median (interquartile range). Categorical variables were expressed as frequencies and percentages. Statistical significance was assumed for p < 0.05.

## Results

The study population included 63 men and 30 women. According to clinical diagnosis, the study population (93 patients) was distributed into the three groups as follows: 43 patients with pleural TB confirmed by culture and/or compatible pleural biopsy specimen (none of them was on treatment), 23 patients with malignancy, and 27 patients with benign nontuberculous effusion (pleural effusion due to an etiology other than TB or malignancy). Within the group with another etiology, 27 patients had pleural effusions associated with bacterial pneumonia or nonspecific pleural effusion, pulmonary embolism, parapneumonic effusions. Age and gender characteristics of pleural and serum groups were presented in Tables 1 and 2. Groups did not differ from each other with respect to smoking habits neither in the pleural measurements group (p = 0.267), nor in the serum measurements group (p = 0.427).

**Table 1:** Pleural adenosine deaminase and interferon-γ levels in patients with tuberculosis, malignancy and non-tuberculosis non-malignancy in the study group

	**Group 1 (Tuberculosis) (n = 43)**	**Group 2 (Malignancy) (n = 23)**	**Group 3 (Non-tuberculosis non-malignancy) (n = 27)**	**p**	
**Age (yrs)**	26 (20–35)	62 (49–75)	67 (44–75)	0.0001	P (1–2) = 0.0001
					P (1–3) = 0.0001
					P (2–3) = 0.907
**Male/female**	30/13	12/11	21/6	0.144	
**Pleural ADA-1 (U/L)**	21.06 (14.55-33.56)	7.48 (5.61-17.16)	14.89 (7.39-21.06)	0.0001	P (1–2) = 0.0001
					P (1–3) = 0.008
					P (2–3) = 0.067
**Pleural ADA-2 (U/L)**	38.00 (29.66-53.15)	8.86 (6.40-12.60)	11.32 (5.80-15.23)	0.0001	P (1–2) = 0.0001
					P(1–3) = 0.0001
					P(2–3) = 0.402
**Pleural total ADA (U/L)**	60.04 (44.66-87.60)	19.78 (16.02-24.43)	23.92 (16.36-40.11)	0.0001	P (1–2) = 0.0001
					P (1–3) = 0.0001
					P (2–3) = 0.170
**Pleural μg/ml (pg/ml)**	185.00 (105.00-460.00)	55.00 (15.00-110.00)	75.00 (36.00-195.00)	0.0001	P (1–2) = 0.0001
					P (1–3) = 0.006
					P (2–3) = 0.110

ADA, Adenosine deaminase; IFN-γ, *Interferon gamma.*

Data are expressed as median (interquartile range).

**Table 2:** Serum adenosine deaminase and interferon-γ levels in patients with tuberculosis, malignancy and non-tuberculosis non-malignancy in the study group

	**Group 1 (Tuberculosis) (n = 23)**	**Group 2 (Malignancy) (n = 11)**	**Group 3 (Non-tuberculosis non-malignancy) (n = 18)**	**p Value**	
Age (yrs)	25 (20–37)	65 (50–68)	67.5 (45–75)	0.0001	P (1–2) = 0.0001
					P (1–3) = 0.0001
					P (2–3) = 0.946
Male/female	15/8	7/4	15/3	0.368	
Serum ADA-1 (U/L)	11.32 (4.43-18.01)	11.52 (8.64-16.24)	10.81 (6.70-14.89)	0.983	
Serum ADA-2 (U/L)	16.54 (12.11-20.77)	11.36 (7.16-20.00)	10.00 (6.59-13.00)	0.004	P (1–2) = 0.068
					P (1–3) = 0.001
					P (2–3) = 0.393
Serum total ADA (U/L)	28.98 (24.51-33.66)	27.39 (15.68-35.14)	21.70 (17.73-27.05)	0.034	P (1–2) = 0.473
					P (1–3) = 0.004
					P (2–3) = 0.605
Serum μg/ml (pg/ml)	75.00 (35.00-160.00)	50.00 (15.00-90.00)	57.50 (30.00-115.00)	0.513	

ADA, Adenosine deaminase; IFN-γ*,* Interferon gamma.

Data are expressed as median (interquartile range).

Comparative characteristics of pleural adenosine deaminase and pleural interferon-γ levels measurements among patients with tuberculosis, with malignancy and with benign non-tuberculosis pleural effusions are presented in Table 1. Serum measurements group included 43 patients with pleural TB, 23 patients with malignant effusions and 27 with benign non-tuberculosis effusions. pADA-1, pADA-2, total pADA, and pIFN-γ levels were significantly higher in patients with pleural TB than in other patients (p < 0.0001).

Comparative characteristics of serum adenosine deaminase, pleural interferon-γ levels among patients with tuberculosis, patients with malignancy and patients with benign non-tuberculosis effusions are presented in Table 2. Serum measurements group included 23 patients with pleural TB, 11 patients with malignant pleural effusion, and 18 patients with benign non-tuberculosis effusions. There were no significant differences in sADA-1 and sIFN-γ values among. sADA-2 and total sADA levels significantly differed between patients with pleural TB and those with benign non-tuberculosis effusions (p = 0.001, and p = 0.004, respectively).

**Figure 1:**
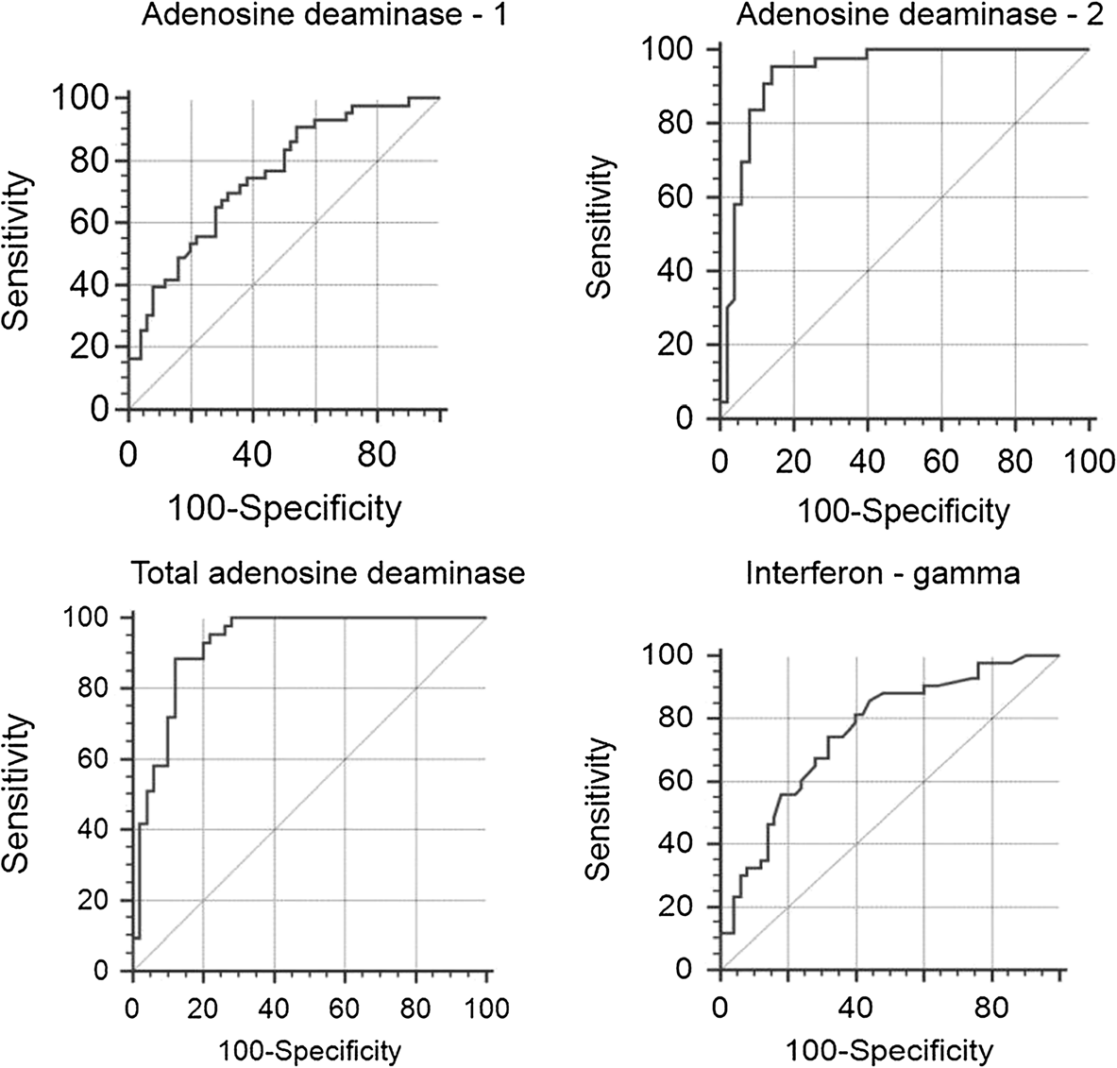
**Receiver operating characteristic (ROC) curves showing the sensitivity and specificity presented in Table **1** at various cut-off values for adenosine deaminase-1, adenosine deaminase-2, total adenosine deaminase, and interferon-gamma in pleural fluid.**

**Figure 2:**
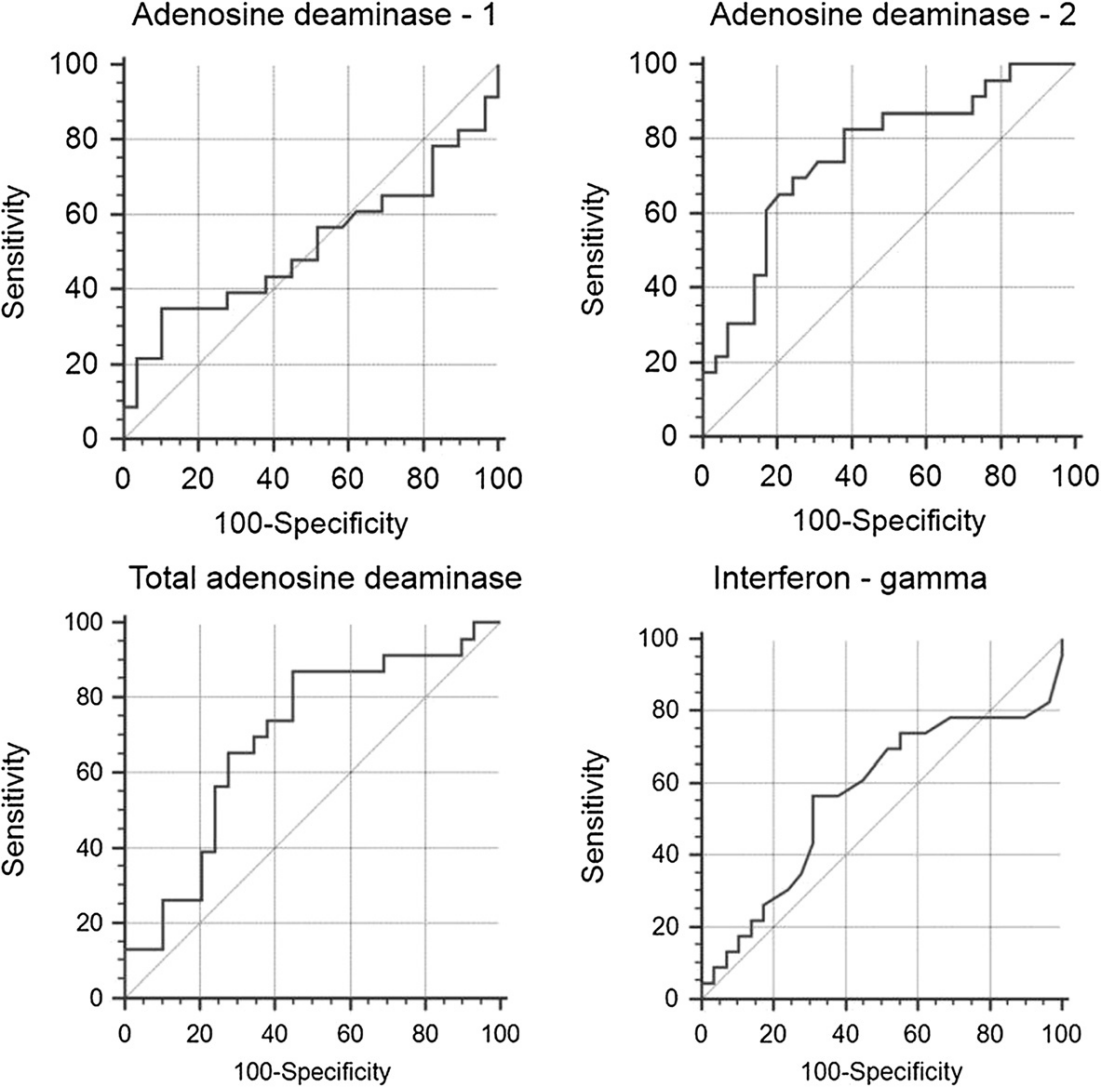
Receiver operating characteristic (ROC) curves showing the sensitivity and specificity at various cut-off values for adenosine deaminase-1, adenosine deaminase-2, total adenosine deaminase, and interferon-gamma in serum.

As for pleural TB receiving operating characteristic (ROC) curves identified the following results (Table 3) (Figures 1 and 2). The best cut-off value for pADA-2 was 20.37 U/L and yielded a sensitivity and specificity of 95.35% and 86%, respectively. Taking a cut-off value of 40.68 U/L for total pADA, the sensitivity and the specificity were found to be 88.37% and 88%, respectively. ROC curve identified 110 U/L as the best cut-off value for pIFN-γ, while the sensitivity and the specificity were found to be 74.42% and 68%, respectively. Finally, the best cut-off value for pADA-1 was 16.8 U/L and yielded a sensitivity and specificity of 69.77% and 68%, respectively.

**Table 3:** Pleural and serum adenosine deaminase and interferon-γ levels evaluated for the diagnostic accuracy of pleural tuberculosis

	**Cut-off (U/L)**	**Sensitivity (%)**	**Specificity (%)**	**PPV (%)**	**NPV (%)**	**AUC ± Sh**	**p Value**
**Pleural ADA-1**	>16.8	69.77	68	65.2	72.3	0.750 ± 0.051	0.0001
**Pleural ADA-2**	>20.37	95.35	86	85.4	95.6	0.936 ± 0.027	0.0001
**Pleural total ADA**	>40.68	88.37	88	86.4	89.8	0.925 ± 0.029	0.0001
**Pleural IFN-γ**	>110	74.42	68	66.7	75.6	0.759 ± 0.051	0.0001
**Serum ADA-1**	≤5.34	34.78	89.66	72.7	63.4	0.515 ± 0.081	0.8536
**Serum ADA-2**	>13.64	69.57	75.86	69.6	75.9	0.759 ± 0.069	0.0002
**Serum total ADA**	>23.07	86.96	55.17	60.6	84.2	0.694 ± 0.074	0.0095
**Serum IFN-γ**	>70	56.52	68.97	59.1	66.7	0.567 ± 0.081	0.4096

ADA, Adenosine deaminase; AUC, Area under the curve; IFN-γ, Interferon gamma; NPV, Negative predictive value; PPV, Positive predictive value.

For pleural TB cases, the area under the curve (AUC) of the ROC curve was highest for pADA-2 (0.936 ± 0.027), followed by total pADA (0.925 ± 0.029), pIFN-γ (0.759 ± 0.051), sADA-2 (0.759 ± 0.069), and pADA-1 (0.750 ± 0.051) (Table 3).

## Discussion

Conventional methods to diagnose pleural TB have proven to be insufficient. Every test that could increase the confidence of resuming or discontinuing antituberculosis therapy is of clinical relevance, especially in endemic areas of the world. Thus, there is a need to develop a diagnostic marker that can offer a rapid and accurate diagnosis. The quantification of IFN-γ and the measurement of ADA have shown high sensitivity and specificity in pleural TB, especially in the areas of high prevalence [12,13]. In this study, we aimed to evaluate and compare the diagnostic efficiency of serum and pleural levels of ADA-1, ADA-2, total ADA, and IFN-γ for the differential diagnosis of pleural TB.

The sensitivity and robustness of ADA activity in the diagnosis of pleural TB, together with its simplicity, speed, and low cost, urge the widespread implementation and routine utilization of the method. However, the cause of increased ADA activity in pleural TB is still uncertain. The suggestion that it is related to increased lymphocyte counts in these effusions appears to have been ruled out by several studies [14,15]. The finding that increased ADA activity in tuberculous pleurisy is due largely to increased activity of the ADA isoenzyme ADA-2, together with the fact that the only cells in which ADA-2 has been found are monocytes/macrophages, led Gakiset al. to attribute increased ADA activity in pleural TB to the stimulation of monocytes/macrophages by live phagocytosed micro-organisms [16]. The unique origin of ADA-2 has been confirmed by Ungereret al*.*, who revealed that the increased ADA activity in pleural TB is due largely to increased ADA-2 activity [17].

A meta-analysis of 31 studies published between 1978 and 2000, which assessed the value of pleural fluid ADA activity in differential diagnosis of pleural TB demonstrated a high sensitivity and specificity of these measurements (92 and 89%, respectively) [18]. In the most recent meta-analysis of studies investigating the use of pleural fluid ADA activity for the diagnostic evaluation of pleural TB, in which a total of 63 studies have been analyzed, sensitivity was estimated at 92% and specificity at 90% [19]. Similar to our study, in over 2/3 of these studies ADA activity was measured with a colorimetric method described by Gusti [8]. However, Zaricet al.found that the diagnostic sensitivity of ADA determination in tuberculous pleural effusion was 89.2%, but the specificity was only 70.4% [20]. One reason for this discrepancy may be the differences in the methodology of ADA activity measurements. Although the cut-off value applied by different authors ranged between 10 and 70 U/L, the mean total pADA cut-off value in 31 analyzed studies was very close to the best discriminating value in our study (41.8 and 40.68 U/L, respectively).

Two molecular forms of ADA, each with its own unique properties, have been identified in humans: ADA-1 and ADA-2 [21]. Each isoform is coded for by a different gene locus [17]. ADA-1 has the highest activity in lymphocytes and monocytes, whereas ADA-2 appears to be originated from monocytes [22]. Ungerer and Grobler identified ADA-2 and ADA-1 in pleural fluid, with ADA-2 being predominant in TB [23]. The latter finding was confirmed by Kurataet al*.*[20]. Ungereret al*.* repeated their results, and showed that parainfective effusions were associated with ADA-1, while tuberculous effusions were associated with the ADA-2 isoenzyme [24]. With a cut-off level of 20.37 U/L estimated from pADA-2 ROC analysis, we found relatively high diagnostic sensitivity and specificity of the test (95.35 and 86%), respectively.

IFN-γ proved to be highly associated with tuberculous etiology of pleural effusion [5]. Redistribution of T and B cells and the stimulation of T cells present in the pleural cavity explain the differences between the concentration of IFN-γ in pleural fluid and peripheral blood. One of the studies demonstrated that median IFN-γ concentration in pleural effusion was over 60 times higher than that in the blood [25]. However, in our study, in patients with TB, pleural IFN-γ concentration was only 2.4 times greater than the serum concentration. The sensitivity and specificity of IFN-γ measurements as a diagnostic marker of pleural TB ranged from 85.7 to 100%, and from 95 to 97%, respectively [12,26]. The methodology of IFN-γ determination used in most of the studies was similar and based on using commercial immunoenzymatic assays (ELISA). In 2007, a meta-analysis which summarized the results of 22 studies evaluating the usefulness of IFN-γ determination for the diagnosis of TPE was published [12]. In this meta-analysis, the mean sensitivity was 89%, while the mean specificity was 97%. However, in our study, we obtained very low sensitivity and specificity percentages when compared to the literature. As for pleural IFN-γ levels, by setting the cut-off value for pIFN-γ concentration at 110 U/L we obtained diagnostic sensitivity of 74.42%, specificity of 68%, negative predictive value of 75.6%, and positive predictive value of 66.7%. As for serum IFN-γ levels, by setting the cut-off value for pμg/ml concentration at 70 U/L we obtained diagnostic sensitivity of 56.52%, specificity of 68.97%, negative predictive value of 66.7%, and positive predictive value of 59.1%. The main reason for the low sensitivity and specificity determined in our study is thought to be due to the small sample size.

ADA and IFN-γ measurements are simple and have the advantage of being a rapid and direct means of detecting M. tuberculosis in pleural fluid, whereas PCR is a more demanding and expensive method . Limitations of our study are the relatively small size of our series and lack of definite criteria for selection of patients for this method. However, we hope that this study will pioneer further studies for the non-invasive differential diagnosis of pleural TB.

## Conclusions

In conclusion, pleural ADA-2 and total ADA activities are sensitive and specific markers of pleural TB. In distinguishing pleural TB, pleural levels of ADA-2 have the highest sensitivity among the different diagnostic parameters and may find a place as a routine investigation in the coming days for early detection of TB. Although these tests may reduce the number of patients referred to more invasive diagnostic procedures, they should not be considered an alternative to biopsy and culture. Cultures of pleural fluid and biopsy specimens have a greater diagnostic yield.

## Competing interest

The authors declare that they have no competing interest.

## Authors’ contributions

All authors read and approved the final manuscript.
